# Primary Care Signals and Diagnostic Pathways Before Incident Heart Failure: A Systematic Scoping Review

**DOI:** 10.1177/21501319261488368

**Published:** 2026-09-09

**Authors:** Waseem Jerjes, Austen El-Osta, Arnoupe Jhass, Adam Taylor, Gabrielle Oh, Azeem Majeed

**Affiliations:** 1 School of Public Health, Faculty of Medicine, 4615 Imperial College London, London, UK; 2 Institute for Health Informatics, 4919 University College London, London, UK

**Keywords:** heart failure, primary care, diagnosis, diagnostic pathways, scoping review, electronic health records

## Abstract

**Introduction:**

Heart failure may be preceded by non-specific symptoms or indicators recorded in primary care, although these records do not necessarily represent heart failure or missed diagnosis. We mapped quantitative evidence on prediagnostic signals, investigation, referral, diagnosis setting and outcomes.

**Methods:**

We conducted a systematic scoping review of quantitative observational studies of incident or newly diagnosed adult heart failure that included a primary care, community or ambulatory first-contact component. Databases were searched from January 2000 to 1 May 2026. Two reviewers independently screened records, extracted data and assessed risk of bias using Joanna Briggs Institute tools. Heterogeneous findings were synthesised narratively and organised by dataset family.

**Results:**

Eleven reports were included as sources of evidence. Most were UK retrospective linked-record cohorts; six reports used overlapping national English data, and five used independent datasets from Portugal, other UK regions and the USA. Recorded symptoms or indicators before diagnosis occurred in 30.0% to 66.5% of patients. Among 80,824 English patients with prior indicators diagnosed in 2015–19, 12.5% had recorded natriuretic peptide testing, 19.8% echocardiography and 34.4% specialist referral in the six months before diagnosis. Hospital or acute-care diagnosis was common and was associated with poorer mortality, admission or cost outcomes. Repeat consultations and clinicians’ responses to recurrence were rarely measured directly.

**Conclusion:**

The evidence describes recorded prediagnostic intervals and pathway gaps, not proven missed or avoidable diagnostic delay. It does not establish repeated or unresolved presentation as a clinical trigger. Primary studies should define recurrence, determine whether earlier records plausibly reflected heart failure, and adjudicate whether repeated presentation identifies preventable diagnostic opportunities.

## Introduction

Heart failure is a common, costly and clinically serious syndrome associated with recurrent hospital admission, reduced quality of life and poor survival. Contemporary population-based evidence shows that outcomes remain poor despite therapeutic advances, with persistent inequalities by sex, socioeconomic status and diagnosis setting. Patients first diagnosed during hospital admission generally have worse outcomes than those diagnosed in the community, while improvements in survival after heart failure diagnosis have been modest compared with other major long-term conditions.^1-5^

The diagnostic pathway for heart failure usually begins in primary care. Patients commonly present with breathlessness, ankle swelling, fatigue, reduced exercise tolerance or overlapping cardiorespiratory symptoms. However, timely recognition in general practice remains difficult. Symptoms are non-specific, multimorbidity is common, and diagnosis depends on access to natriuretic peptide testing, echocardiography and specialist review. Qualitative evidence and clinician surveys describe continuing diagnostic uncertainty, difficulty applying guidelines to complex older patients, limited access to diagnostic services and inconsistent coordination between primary and specialist care.^6,7^ Wider evidence also shows that guideline-recommended testing and referral are incompletely implemented in routine primary care.^8-10^

Linked-data studies have begun to quantify these problems. UK evidence shows that management and survival have changed over time, but hospital-based diagnosis remains common and survival remains poor.^11^ Studies of route to diagnosis, practice variation, guideline adherence, diagnostic testing and regional pathways show that only a minority of symptomatic patients follow a guideline-aligned route, while recorded intervals between earlier symptoms or indicators and diagnosis may extend for months or years.^12-18^ Such intervals describe chronology; they do not by themselves establish a missed diagnosis or avoidable delay.

Diagnosis setting is clinically important. Studies comparing community and hospital pathways have associated hospital-based diagnosis with worse short-term outcomes and greater healthcare cost, while US data show that many patients diagnosed in acute care had potential heart failure symptoms documented in outpatient records beforehand.^19,20^ More recent work has identified candidate indicators of later diagnosis and highlighted the risks of both overdiagnosis and underdiagnosis in primary care.^21-25^ These studies also emphasise that case ascertainment is heterogeneous and that breathlessness, oedema and fatigue are not specific to heart failure.

The literature is clinically important but fragmented. Studies examine route to diagnosis, testing, referral or diagnosis setting, but few identify repeated related consultations in the same patient or clinicians’ response to recurrence. We treated repeated or unresolved presentation as an a priori hypothesis, not an established exposure. This review mapped evidence on recorded primary care signals, escalation, diagnosis setting and outcomes, asking whether studies supported that hypothesis or instead defined an evidence gap.

## Methods

### Study Design and Reporting

We conducted a systematic scoping review of quantitative observational studies examining recorded primary care signals, diagnostic escalation, potential missed opportunities and route to incident heart failure diagnosis in adults. The protocol was developed prospectively and registered with PROSPERO (CRD420261366975), and the PROSPERO record was revised to reflect the final scoping-review design. The review is reported using PRISMA-ScR, with narrative synthesis and structured evidence mapping used to summarise heterogeneous evidence.

The registered protocol described a systematic review with exploratory random-effects meta-analysis, sensitivity analyses and a GRADE-informed assessment. These were not undertaken because exposures, outcomes, time windows and data sources were too heterogeneous, and several reports could overlap. We therefore used a scoping approach, grouped reports by dataset family and revised the PROSPERO record to reflect these methodological amendments. The population, incident-diagnosis focus and pathway-based eligibility criteria were retained.

### Eligibility Criteria

Studies were eligible if they included adults (≥18 years) with incident or newly diagnosed heart failure and incorporated a primary care, general practice, family practice, community or ambulatory first-contact component. To be eligible, studies had to reconstruct at least one component of the individual patient pathway before heart failure diagnosis. Specifically, studies were required to report one or more clinical signals individually recorded before diagnosis (for example symptoms, signs, biomarkers, prescribing changes or healthcare contacts) together with a subsequent linked diagnostic action, investigation, referral, recorded interval, route to diagnosis or diagnosis setting forming a reconstructed pathway that allowed the prediagnostic pathway to be examined.

Studies that reported only diagnosis setting, prognosis, healthcare utilisation, survival, service performance or postdiagnosis management, without reconstructing the patient pathway before diagnosis, were excluded. Aggregate healthcare-contact trajectories without signal-specific diagnostic response, and isolated test-to-diagnosis intervals reported within prognostic analyses, were not considered pathway reconstruction. Where diagnosis setting was the principal analytic focus, studies were eligible only if diagnosis setting was linked to downstream clinical or economic outcomes or to variation at the primary-care clinician or general-practice level; health-system or facility-level variation alone was insufficient. Hospital-only studies without an identifiable community pathway, screening studies in asymptomatic populations, paediatric studies, editorials, commentaries, protocols, case reports and very small case series were also excluded. Retrospective and prospective cohort studies, routinely collected database studies, registry-linkage studies, claims-based studies and analytical cross-sectional studies were eligible where these criteria were met. Qualitative and consensus studies were retained only to inform background interpretation and were not included in the quantitative synthesis.

### Heart Failure Definition and Interpretive Terminology

Heart failure was accepted as defined by each study. Definitions included primary care or hospital diagnostic codes, linked primary-secondary care algorithms, claims-based codes, first heart failure hospitalisation, echocardiography-linked definitions and echocardiography plus loop-diuretic criteria. We extracted each ascertainment method and any reported phenotype or ejection fraction; these data were unavailable or incomplete in several large routine-data studies.

To avoid attributing causality to routine records, we distinguished recorded prediagnostic interval, potential diagnostic opportunity, adjudicated missed opportunity and proven avoidable delay. A recorded interval was elapsed time between a coded signal and diagnosis. A potential opportunity required an earlier encounter meriting review; an adjudicated missed opportunity required prespecified clinical criteria; and avoidable delay required evidence that earlier action was feasible and would have changed timing. Repeated or unresolved presentation required at least two related contacts or documented persistence in the same patient. We used each term only when supported by the source study.

### Information Sources and Search Strategy

MEDLINE through PubMed, Embase through Ovid, Scopus and Web of Science Core Collection were searched from 1 January 2000 to 1 May 2026. Database-specific strategies combined controlled vocabulary and free text for heart failure, primary or ambulatory care, and prediagnostic presentation, investigation, referral, missed opportunity, diagnostic delay or route to diagnosis, with symptom terms added where appropriate. Eligibility was restricted to human and English-language records. Reference lists and forward citations were checked. Complete reproducible strategies are provided in Supplementary Appendix 1.

### Study Selection and Data Extraction

All retrieved records were imported into reference-management software and de-duplicated. Two reviewers independently screened titles and abstracts and then assessed potentially eligible full-text reports. Disagreements were resolved through discussion and, where required, consultation with a third reviewer. Throughout this review, “report” refers to an individual publication assessed for inclusion, whereas “study” refers to the underlying investigation or dataset from which one or more reports were derived.

Two reviewers independently extracted author, year, country, setting, design, data source, study period, sample size, heart failure definition, phenotype availability, primary care component, recorded symptoms or indicators, investigations, referral, route and setting of diagnosis, interval measures, admissions, mortality, costs, effect estimates and limitations. Dataset identifiers, observation periods and cohort definitions were extracted specifically to assess possible population overlap.

### Risk of Bias and Synthesis

Methodological quality was assessed independently by two reviewers using the Joanna Briggs Institute checklist appropriate to each design, usually the cohort checklist. Items were rated yes, no, unclear or not applicable; disagreements were resolved by discussion or a third reviewer. Item-level judgements and rationales are reported in Supplementary Appendix 2. Overall descriptors aided narrative interpretation only and were not calculated from a threshold or used for exclusion.

Synthesis was organised first by dataset family and then across four domains: recorded signals, diagnostic escalation, diagnosis setting and outcomes. Concordant findings from overlapping datasets were not treated as independent replication. The harvest plot shows whether each report contributed to a domain; bar height indicates sample-size category and fill or outline indicates overall risk of bias, not effect magnitude. The timeline displays reported intervals, not proven delay. Formal GRADE ratings were not applied because there was no common outcome-level effect estimate; confidence was considered narratively through directness, consistency, overlap and risk of bias. Ethics approval was not required because only published, non-identifiable data were used.

## Results

### Study Selection

**Figure 1.**
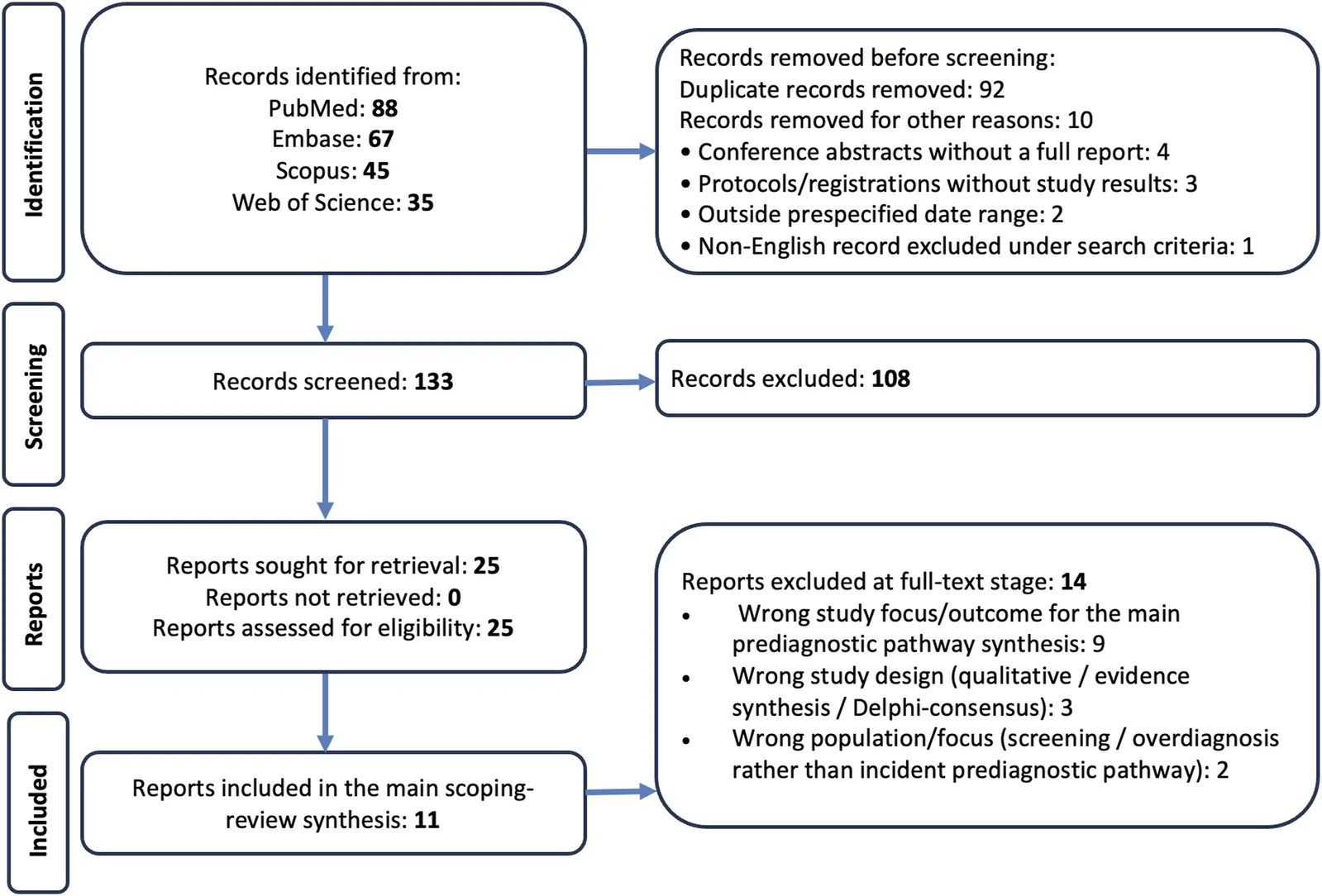
PRISMA-ScR flow diagram for identification, screening, eligibility assessment and inclusion in the scoping review

The search identified 235 records: 88 from PubMed, 67 from Embase, 45 from Scopus and 35 from Web of Science. Before screening, 92 duplicates and 10 other records were removed: conference abstracts without a full report (n=4), protocols or registrations without results (n=3), records outside the date range (n=2) and one non-English record excluded under the search criteria. The remaining 133 records were screened and 108 excluded. Twenty-five reports were assessed at full text. Fourteen reports were excluded after full-text assessment because they did not meet the predefined pathway-focused eligibility criteria. Nine reported diagnosis setting, prognosis, diagnostic testing, service performance or postdiagnosis outcomes without reconstructing the patient pathway before diagnosis; three had an ineligible study design; and two addressed an ineligible population or research focus. Eleven reports entered the synthesis and were organised into six dataset families according to their underlying study populations (Figure 1).

### Study Characteristics, Ascertainment and Dataset Families

The 11 included reports, published between 2018 and 2025, were predominantly retrospective linked-record cohort analyses. Most were from the UK, with additional evidence from Portugal and the USA. Samples ranged from 2436 first heart failure hospitalisations to 959,438 claims-based incident diagnoses. Characteristics and dataset families are summarised in Table 1.

**Table 1.** Characteristics of Included Reports, Dataset Families and Possible Population Overlap

Study	Country/setting	Design and data source	Analytic sample	Primary care/community diagnostic component	Main pathway/delay concept	Main outcomes relevant to this review	Dataset family/possible overlap
**Bottle et al. 2018 (Heart)**	England, national	Observational linked-data study using CPRD primary care records linked to HES and ONS	36,748	Primary care consultations with recorded HF symptoms in the 5 years before diagnosis	Routes to diagnosis; alignment with NICE-recommended investigation/referral pathway	Prior symptom-recorded consultations; proportion following guideline-aligned pathway; hospital vs primary care first recording	CPRD-derived national English family; possible overlap with other CPRD reports
**Bottle et al. 2018 (Open Heart)**	England, national	Retrospective cohort using CPRD-linked primary and secondary care data	13,897	Symptomatic patients presenting to GPs with breathlessness, fatigue, or ankle swelling	Initial management after symptomatic primary care presentation; full/partial/no NICE pathway	Emergency HF admission and all-cause death by pathway group	CPRD-derived family; same 13,897-patient cohort as Kim 2019; related to Hayhoe 2019
**Bottle et al. 2022**	UK primary care	Observational comparison of two CPRD cohorts 10 years apart	5,966 (2001/02) and 12,827 (2011/12); symptomatic subgroup 9,963	GP-recorded symptoms before diagnosis and subsequent diagnostic work-up	Change over time in management, guideline compliance, and diagnosis setting	Long-term survival; echocardiography; BNP testing; specialist referral; prescribing trends	CPRD-derived family; related national data and partly overlapping calendar period
**Kim et al. 2019**	England, primary care	Retrospective cohort study using primary care electronic records	13,897	Patients with symptoms suggestive of HF managed in general practice	Route to HF diagnosis; adherence to NICE-recommended pathway; practice variation	Adherence to recommended route; association with further consultation and patient/practice factors	CPRD-derived family; same 13,897-patient cohort as Bottle 2018 (Open Heart)
**Hayhoe et al. 2019**	England, national	Observational linked-data study using CPRD linked to HES/ONS	42,403 total; 16,597 with suggestive symptoms	Recorded breathlessness, fatigue, or ankle swelling in primary care before diagnosis	Time from symptom presentation to investigation, referral, diagnosis, and treatment	Median time to NP/echo, specialist referral, diagnosis, and HF medication	CPRD-derived family; related 2010–2013 national cohort
**Ferreira et al. 2023**	Portugal, large healthcare organisation	Cohort study using electronic health records	2,436 first HF hospitalisations; 720 with prior signs/symptoms	PCP-level records of HF signs/symptoms before first HF hospitalisation	Missed opportunities for diagnosis/management before first hospital admission	Adequate vs inadequate follow-up/work-up after symptom recording	Independent Portuguese healthcare-organisation dataset
**Migas et al. 2024**	UK, Salford urban linked-data population	Retrospective cohort using linked primary and secondary care data	3,227	Primary and secondary care diagnostic pathway before and around diagnosis	Diagnostic pathways, testing, specialist access, and missed opportunities	NP testing, echocardiography, outpatient cardiology access, survival	Independent Salford linked-data dataset
**Lawson et al. 2025**	England, national	Retrospective CPRD data-linkage cohort	412,173	Primary care HF indicators (breathlessness, ankle swelling, loop diuretic use) up to 5 years before diagnosis	Early identification potential, diagnostic delay, investigation rates, inequalities, diagnosis setting	Lag time to diagnosis; natriuretic peptide testing; echocardiography; specialist review; inpatient vs outpatient diagnosis; 1-year survival	CPRD-derived family; national 2000–2021 data encompass periods used by earlier reports
**Bachtiger et al. 2023**	North West London	Retrospective cohort with propensity-matched analysis using Discover linked primary/secondary care and cost data	34,208 total	Community pathway defined by GP or outpatient diagnosis versus hospital admission pathway	Route to index HF diagnosis by community vs hospital setting	All-cause mortality and longitudinal healthcare cost by diagnosis pathway	Independent North West London Discover dataset
**Wang et al. 2025**	Tayside, Scotland	Population cohort using linked electronic health records and echocardiography data	5,223 total; 4,231 HF	Outpatient diagnosis via echocardiography/loop diuretic criteria versus inpatient HF diagnosis	Inpatient vs outpatient de novo diagnosis across EF spectrum	Cardiovascular death or HF hospitalisation within 12 months; effect of discharge GDMT in inpatients	Independent Tayside echocardiography-linked dataset
**Sandhu et al. 2021**	USA, national insured population	Retrospective claims-based cohort	959,438	Outpatient primary care visits and coded symptoms before acute-care diagnosis	Acute care versus outpatient diagnosis setting; prior outpatient symptoms; clinician variation	Proportion diagnosed in acute care; prior symptoms in 6 months; predictors of acute-care diagnosis	Independent US claims dataset

CPRD, Clinical Practice Research Datalink; HES, Hospital Episode Statistics; ONS, Office for National Statistics; HF, heart failure; NP, natriuretic peptide; BNP, brain natriuretic peptide; GDMT, guideline-directed medical therapy; EF, ejection fraction; NICE, National Institute for Health and Care Excellence. Reports within the CPRD-derived family were treated as potentially overlapping and were not counted as independent replications.

Six reports used national English CPRD-derived data and could overlap at patient or practice level.^11-14,18,25^ The two 13,897-patient analyses used the same 2010-2013 cohort, while other reports used related linkages and periods. We treated these six reports as one evidence family rather than independent replications. The other five studies used independent datasets from Portugal, Salford, North West London, Tayside and the USA.^4,16,17,19,20^

Heart failure ascertainment was heterogeneous. Studies used linked primary care and hospital codes, first heart failure hospitalisation, specialist diagnosis, claims-based ICD codes or echocardiography-linked algorithms. Wang and colleagues combined hospitalisation, echocardiographic evidence and loop-diuretic prescribing, enabling analysis across ejection-fraction groups. Migas and colleagues classified ejection fraction where available, whereas several national primary care studies lacked systematic phenotype data.

### Recorded Primary Care Signals Before Diagnosis

**Figure 2.**
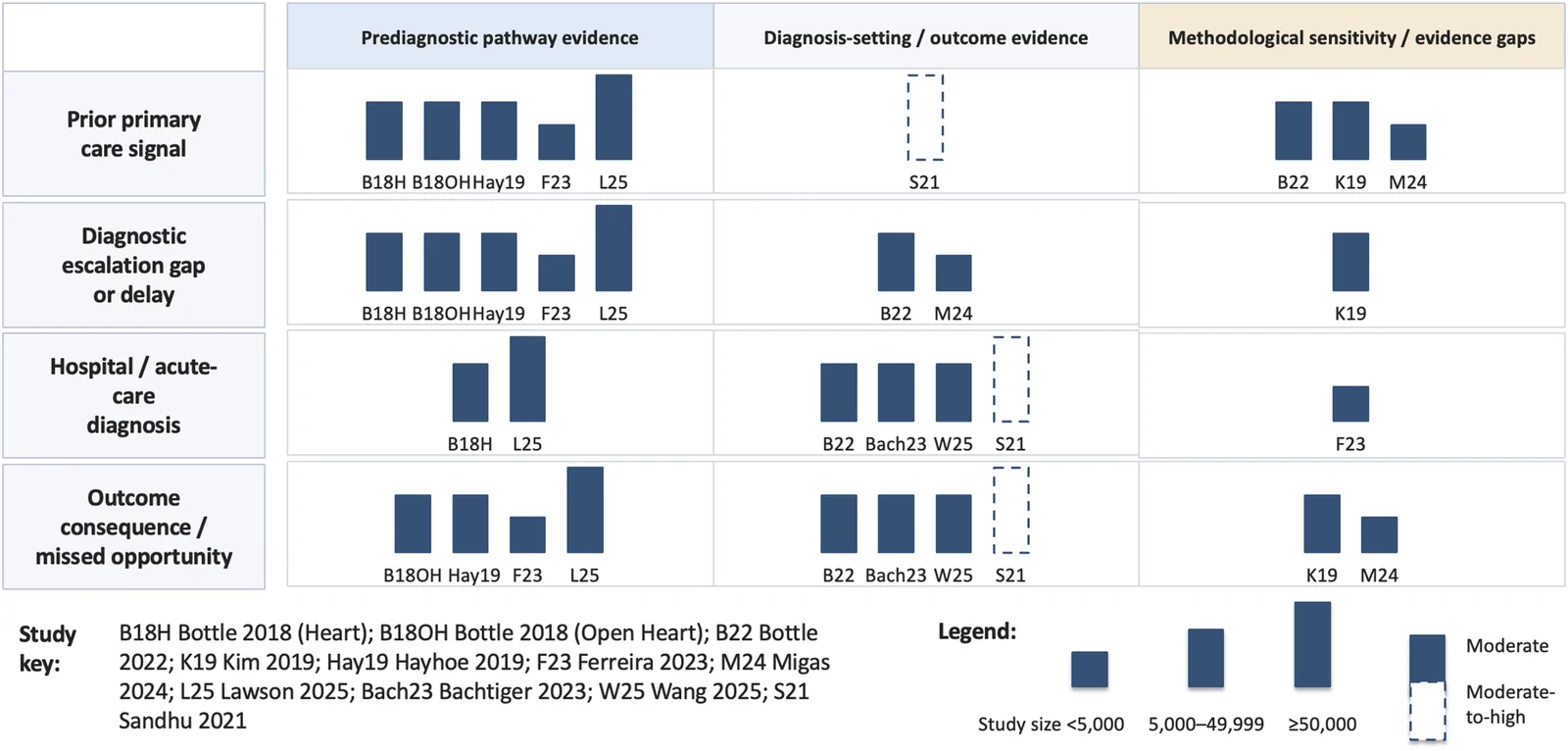
Harvest plot of evidence domains across included reports. Bar height represents total study sample-size category (<5,000; 5,000–49,999; ≥50,000), while solid fill and dashed outline denote moderate and moderate-to-high overall risk of bias, respectively. Bar position indicates contribution to an evidence domain, not effect size; there is no continuous y-axis

Symptoms or indicators were commonly recorded before diagnosis, although the studies did not establish that the earliest record represented heart failure. In the national English route-to-diagnosis study, 15,057 of 36,748 patients (41.0%) had at least one key symptom recorded in primary care. The contemporary English cohort recorded prior indicators in 274,228 of 412,173 patients (66.5%). Corresponding proportions were 30% before first hospitalisation in Portugal and 46.4% among US patients diagnosed in acute care who had preceding outpatient primary-care visits. Table 2 and Figure 2 map these findings. No included study validated the incremental diagnostic value of recurrence beyond individual symptoms.

**Table 2.** Quantitative Findings From Included Reports, Organised by Dataset Family

Study	Prediagnostic primary care signal	Investigation/referral gap in the six months before diagnosis	Diagnosis setting/outcomes	Dataset family
**Bottle et al. 2018 (Heart)**	15,057/36,748 (41.0%) had a primary care consultation with at least one key HF symptom recorded before diagnosis	Only 23.6% of symptomatic patients followed a NICE-aligned pathway; 44% had no echocardiogram, natriuretic peptide test, or referral	79.2% were first recorded in hospital	CPRD
**Bottle et al. 2018 (Open Heart)**	Included 13,897 patients presenting to GPs with breathlessness, fatigue, or ankle swelling	Within 6 months, only 7% completed the full NICE pathway; 18.6% followed it partially	Full or partial NICE-pathway completion was associated with lower crude HF-admission risk than medication-only or no-pathway care	CPRD
**Bottle et al. 2022**	Symptomatic subgroup: 9,963 patients with HF-specific symptoms before diagnosis	Echocardiography after symptom presentation increased from 8.3% to 19.3% over 10 years; specialist referral from 7.2% to 24.6%; BNP testing remained low	5-year survival overall remained poor (∼40%); survival improved mainly in primary care-diagnosed patients (5-year survival 46.0% to 57.4%) but not in hospital-diagnosed patients (33.9% to 32.6%)	CPRD
**Kim et al. 2019**	Further consultation for suggestive symptoms was associated with following the recommended route	Only 7% followed the recommended route to diagnosis	Route to diagnosis varied significantly between GP practices	CPRD
**Hayhoe et al. 2019**	16,597 patients presented in primary care with suggestive symptoms	39% had recorded NP testing or echocardiography; 36% had specialist referral	Median time from symptom(s) to investigation 292 days, to referral 236 days, to diagnosis 972 days, and to HF medication 803 days	CPRD
**Ferreira et al. 2023**	720/2436 (30%) had HF signs/symptoms recorded before first HF hospitalisation; 94% were recorded within 6 months before admission	Among symptomatic patients not already adequately managed, 155/310 **(50%)** had inadequate follow-up	Study applied its own missed-opportunity definition before first HF hospitalisation	Portugal independent
**Migas et al. 2024**	Cohort of 3,227 patients with HF diagnosed in linked UK primary/secondary care data	At baseline, NP data were available in 18.3% and echocardiographic report data in 81.2%; baseline included measurements before or within 90 days after diagnosis. Before diagnosis, 65.0% had cardiology-led outpatient clinic access	5-year crude survival was poor across EF groups: 37.8% (HFpEF), 42.3% (HFrEF), 45.5% (HFmrEF)	Salford independent
**Lawson et al. 2025**	274,228/412,173 (66.5%) had previous HF indicators before diagnosis	Among 80,824 with prior indicators diagnosed in 2015–19, only 12.5% had natriuretic peptide testing, 19.8% echocardiography, 34.4% specialist referral, and 52.3% no diagnostic investigations recorded in primary care	Median lag worsened from 16.4 to 35.4 months; inpatient diagnosis rose from 33.9% to 46.8%; delays, absent investigation, and inpatient diagnosis were each associated with higher mortality	CPRD
**Bachtiger et al. 2023**	Compared community versus hospital pathway to index HF diagnosis in 34,208 patients	Not a testing study primarily, but community diagnosis may represent earlier recognition before admission	68.2% diagnosed through hospital pathway; at 24 months, community diagnosis had lower death rate than hospital diagnosis (rate ratio 0.780, 95% CI 0.722 to 0.841); hospital-pathway diagnosis incurred £2485 extra longitudinal cost per patient	North West London independent
**Wang et al. 2025**	Compared inpatient versus outpatient de novo HF diagnosis in 4,231 HF patients with echo linkage	Compares inpatient with outpatient first diagnosis; does not adjudicate an earlier missed opportunity	51.3% diagnosed as inpatients; primary outcome occurred in 1,193 of 4,231 patients; inpatient diagnosis was associated with a higher adjusted risk of cardiovascular death or HF hospitalisation (aHR 1.62, 95% CI 1.39 to 1.90)	Tayside independent
**Sandhu et al. 2021**	Among acute-care-diagnosed patients with outpatient primary-care visits between six months and seven days before diagnosis, 46.4% had potential HF symptoms documented	Not focused on testing rates, but shows many acute-care diagnoses were preceded by outpatient symptom signals	38% of incident HF was diagnosed in acute care; odds of acute-care diagnosis increased by 3.2% annually; clinician-level adjusted acute-care diagnosis rates varied substantially (IQR 24.0%–38.9%)	US claims independent

HF, heart failure; NP, natriuretic peptide; BNP, brain natriuretic peptide; NICE, National Institute for Health and Care Excellence; EF, ejection fraction; aHR, adjusted hazard ratio. Hayhoe interval estimates use outcome-specific subsets: investigation, n=6,464; referral, n=6,043; diagnosis and medication, n=8,913.

### Diagnostic Escalation

Recorded investigation and referral were frequently incomplete. In the English route-to-diagnosis study, 23.6% of symptomatic patients followed a NICE-aligned pathway and 44% had no echocardiogram, natriuretic peptide test or referral recorded. In related analyses, 7% completed the recommended route, with marked practice variation. Hayhoe and colleagues recorded natriuretic peptide testing or echocardiography in 39% and specialist referral in 36%. Among 80,824 patients with prior indicators diagnosed in 2015–19 in Lawson and colleagues’ cohort, 12.5% had recorded natriuretic peptide testing, 19.8% echocardiography and 34.4% specialist referral in the six months before diagnosis; 52.3% had none of these recorded.

### Recorded Intervals and Diagnosis Setting

**Figure 3.**
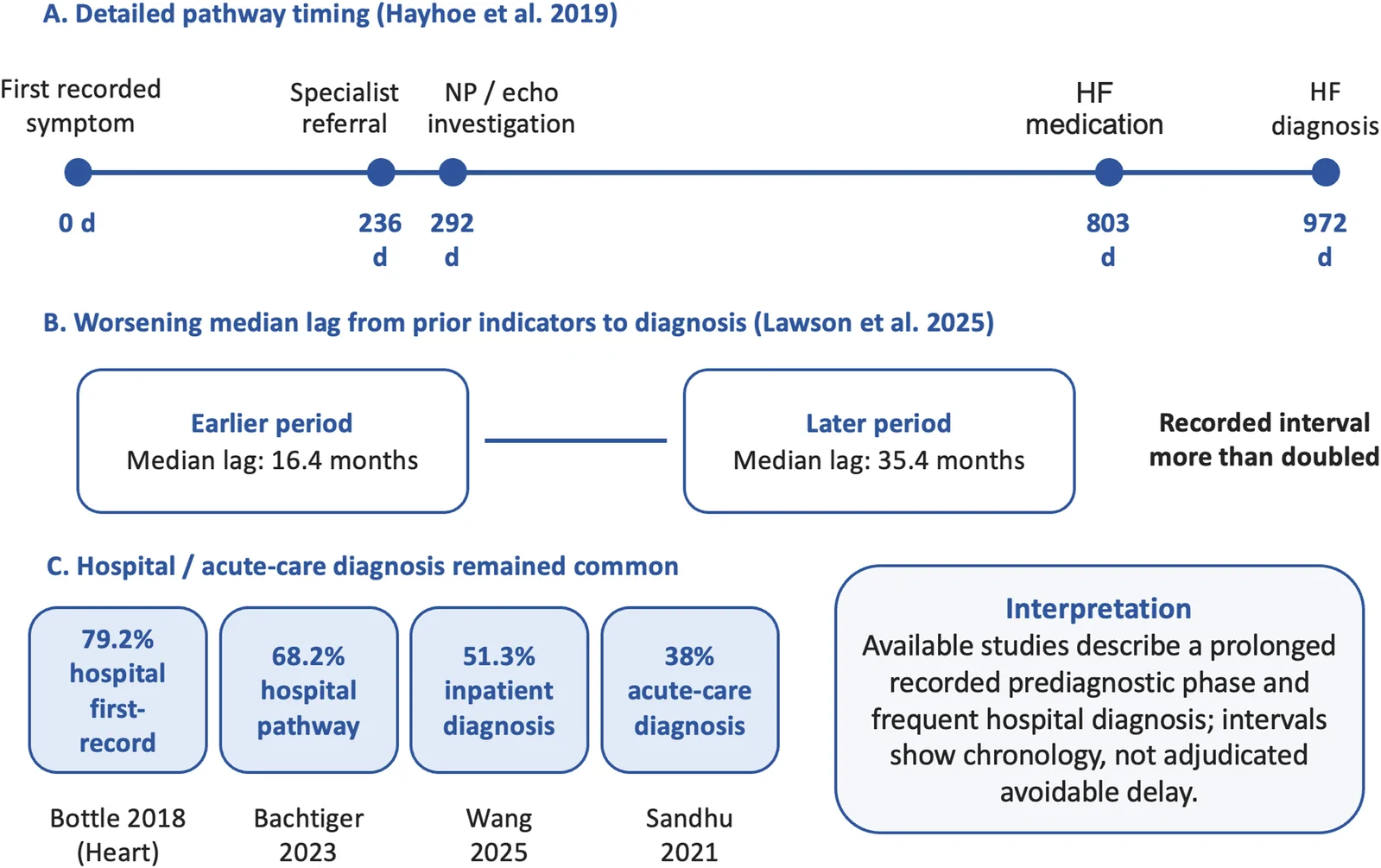
Reported prediagnostic intervals and diagnosis settings across included studies. The first coded symptom or indicator was not adjudicated as the onset of heart failure; intervals describe recorded chronology, not proven diagnostic delay. Hayhoe interval estimates use outcome-specific subsets: investigation, n=6,464; referral, n=6,043; diagnosis and medication, n=8,913.

Hayhoe and colleagues reported median intervals from the first coded suggestive symptom to investigation of 292 days, referral of 236 days, heart failure medication of 803 days and diagnosis of 972 days. The starting code may have reflected another condition or predated clinically recognisable heart failure, so these values should not be read as proven diagnostic delay. Lawson and colleagues reported that the median interval from a first recorded indicator to diagnosis increased from 16.4 months in 2000-04 to 35.4 months in 2015-19 (Figure 3).

Hospital or acute-care diagnosis remained common. Bottle and colleagues found that 79.2% of diagnoses were first recorded in hospital; corresponding estimates were 68.2% for a hospital pathway in North West London, 51.3% for inpatient diagnosis in Tayside and 38% for acute-care diagnosis in the US claims cohort. These independent datasets support the finding that hospital diagnosis is frequent, although definitions and health systems differed.

### Outcome Associations

Several studies associated pathway completion or diagnosis setting with outcomes. In the Open Heart cohort, full or partial NICE-pathway completion was associated with lower crude heart failure admission risk than medication-only or no-pathway care. Survival improved mainly among primary care-diagnosed patients in Bottle et al. 2022. Community diagnosis in North West London was associated with lower mortality and £2485 lower longitudinal cost than a hospital pathway. Inpatient diagnosis in Tayside was associated with higher adjusted risk of cardiovascular death or heart failure hospitalisation, while Lawson and colleagues associated longer intervals, absent recorded investigation and inpatient diagnosis with higher mortality.

### Risk of Bias and Overall Evidence Map

Most studies were judged at moderate risk of bias; the US claims study was moderate to moderately high risk (Table 3). Common concerns were incomplete symptom recording, exposure or pathway misclassification, residual confounding and heterogeneous heart failure ascertainment. Item-level judgements are provided in Supplementary Appendix 2.

**Table 3.** Overall Risk-of-bias Summary of Included Reports; Item-Level Judgements are Provided in Supplementary Appendix 2

Study	Design	JBI tool	Overall judgement	Main concerns driving judgement
**Bottle et al. 2018 (Heart)**	Retrospective linked cohort	JBI cohort	Moderate	Symptom under-recording in routine primary care data; pathway misclassification; residual confounding
**Bottle et al. 2018 (Open Heart)**	Retrospective cohort	JBI cohort	Moderate	Confounding by indication between pathway groups; exposure classification based on coded management; selected symptomatic cohort
**Bottle et al. 2022**	Retrospective cohort comparison of two CPRD eras	JBI cohort	Moderate	Secular changes in coding/recording over time; cohort comparability; incomplete phenotype detail
**Kim et al. 2019**	Retrospective primary care cohort	JBI cohort	Moderate	Selected symptomatic sample; routine coding dependence; practice-level confounding
**Hayhoe et al. 2019**	Retrospective linked cohort	JBI cohort	Moderate	First coded symptom used as start point; possible under-recording of symptoms/investigations; residual confounding
**Ferreira et al. 2023**	Retrospective EHR cohort	JBI cohort	Moderate	Restricted to first HF hospitalisation population; local health-system data; missed-opportunity definition depends on recorded work-up
**Migas et al. 2024**	Retrospective linked regional cohort	JBI cohort	Moderate	Single urban population; incomplete NP/EF capture; potential referral/access bias
**Lawson et al. 2025**	National retrospective data-linkage cohort	JBI cohort	Moderate	Routine-data exposure misclassification; symptom/indicator under-recording; residual confounding despite extensive adjustment
**Bachtiger et al. 2023**	Retrospective propensity-matched cohort	JBI cohort	Moderate	Residual severity confounding despite matching; route-to-diagnosis classification may reflect stage as well as setting
**Wang et al. 2025**	Population cohort with echo linkage	JBI cohort	Moderate	Different inpatient vs outpatient case definitions; possible stage/severity imbalance; observational treatment comparisons
**Sandhu et al. 2021**	Retrospective claims-based cohort	JBI cohort	Moderate to moderately high	Claims-based symptom capture; limited clinical granularity; insured US population reduces transferability to UK primary care

**Figure 4.**
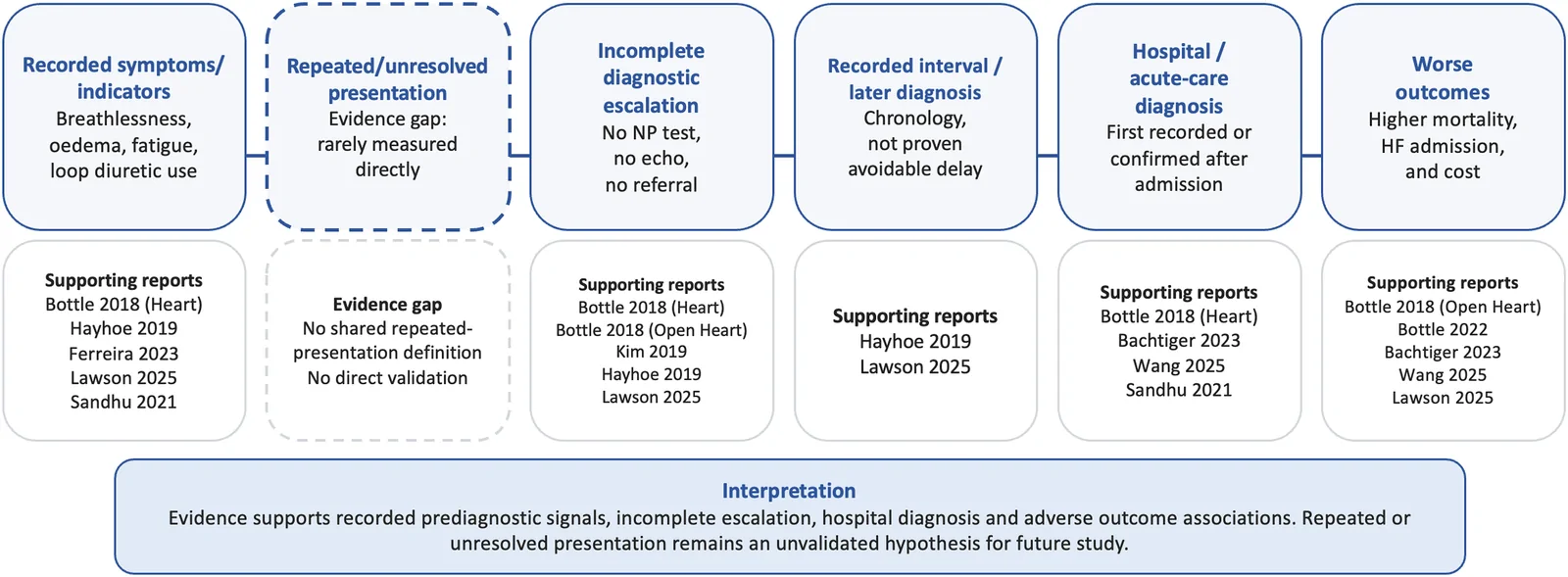
Observed pathway evidence and the unresolved repeated-presentation hypothesis. Solid boxes summarise evidence reported in included studies; the dashed box identifies an evidence gap rather than a validated clinical trigger

Within the CPRD family, reports consistently described prior recorded indicators and incomplete investigation, but their overlap limits claims of replication. Independent datasets supported frequent hospital or acute-care diagnosis and adverse outcome associations. No evidence family validated a recurrent-presentation trigger or adjudicated clinicians’ responses to recurrence. Figure 4 therefore separates observed pathway features from this unresolved evidence gap, and table 4 defines the evidential status of key terms.

**Table 4.** Definitions and Evidential Status of Prediagnostic Pathway Concepts Used in This Review

Term	Operational meaning in this review	Evidence in included studies	Permitted interpretation
Recorded prediagnostic signal	A symptom, prescription, test result or other indicator coded before incident heart failure diagnosis	Commonly reported; prevalence varied by source and definition	Shows that a record preceded diagnosis; does not establish that heart failure was present
Recorded prediagnostic interval	Elapsed time between a coded signal and the recorded diagnosis	Reported by Hayhoe and Lawson using study-specific starting points	Describes chronology, not proven missed or avoidable delay
Potential diagnostic opportunity	An earlier encounter that might merit retrospective clinical review	Suggested indirectly in several pathway studies	A hypothesis requiring patient-level review and adjudication
Adjudicated missed opportunity	An earlier encounter judged against prespecified clinical criteria at which indicated action was not taken	Rare and dependent on study-specific definitions	May support a missed-opportunity claim only within the study definition
Avoidable diagnostic delay	A delay shown to have been preventable, with feasible earlier action likely to alter timing	Not established by the included studies	Must not be inferred from elapsed time alone
Repeated or unresolved presentation	At least two related contacts or documented persistence in the same patient	Rarely measured directly; no shared threshold or adjudication of clinician response	Evidence gap and research hypothesis, not a validated clinical trigger

## Discussion

### Principal Findings

This scoping review found that recorded symptoms or indicators commonly preceded heart failure diagnosis, documented investigation and referral were often incomplete, hospital or acute-care diagnosis was frequent, and diagnosis setting or pathway completion was associated with mortality, admission or cost outcomes.^4,12,14,16,18-20,25^ However, repeat consultations were seldom defined or measured at patient level, and the studies did not adjudicate clinical responses to recurrence. The review therefore does not establish repeated or unresolved presentation as a diagnostic-safety pattern; it identifies this as a hypothesis requiring direct primary research.

### Interpretation

The review brings together evidence streams that are usually considered separately. The review deliberately focused on studies that reconstructed the sequence of events before diagnosis rather than studies describing diagnosis setting, prognosis or healthcare utilisation alone. This distinction explains the exclusion of several otherwise relevant epidemiological studies that did not permit reconstruction of an individual prediagnostic pathway.

Route-to-diagnosis studies record earlier symptoms or indicators; testing studies describe incomplete natriuretic peptide testing, echocardiography or referral; and diagnosis-setting studies show frequent hospital diagnosis.^4,8-14,17-20^ Taken together, these observations describe a possible pathway from recorded signals to incomplete escalation and later hospital diagnosis. They were not always measured in the same patient, however, and should not be interpreted as a demonstrated causal sequence.

Qualitative evidence provides a plausible context for these findings. General practitioners describe uncertainty when symptoms are non-specific, difficulty applying pathways in older people with multimorbidity, variable access to echocardiography and weak coordination with specialist care.^6,7^ The quantitative studies show where those pressures may appear in records, but they do not reveal the reasoning behind individual decisions.

The review began with the hypothesis that recurrence or non-resolution might identify diagnostic risk. Existing studies support two prerequisites for that hypothesis: earlier signals were often recorded, and diagnostic escalation was sometimes absent or incomplete. Two overlapping reports measured further consultations for suggestive symptoms, but neither established whether recurrence represented a missed diagnostic opportunity.^13,25^ The hypothesis is therefore neither validated nor disproved by this review; the evidence is indirect.

Long recorded intervals require caution. The 972-day median began with the first coded symptom, not adjudicated onset of heart failure.^14^ Breathlessness, oedema and fatigue are common in multimorbidity and may reflect respiratory, renal, vascular or functional conditions.^6,7,22,23^ Lawson and colleagues’ increase from 16.4 to 35.4 months similarly describes an interval from a recorded indicator, not necessarily avoidable delay.^18^ These findings justify closer study of earlier encounters, not retrospective attribution of every interval to missed diagnosis.

Dataset overlap also changes the weight placed on apparent consistency. Six national English reports formed one CPRD-derived family, including two analyses of the same 13,897-patient cohort. Their findings are valuable but cannot be counted as six independent confirmations. The five independent datasets more securely support the frequency of hospital or acute-care diagnosis and its association with poorer outcomes; they provide much less direct evidence about repeated presentation or primary care decision-making.

### Strengths and Limitations

The principal strength is the systematic mapping of a fragmented literature across recorded signals, investigation, referral, diagnosis setting and outcomes. Grouping by dataset family, defining interpretive terms, and using tables and figures that distinguish evidence from inference reduce the risk of overstatement. The harvest plot and timeline summarise heterogeneous evidence without implying a pooled effect.

The review also has important limitations. Routine records may under-document symptoms, investigations performed outside the captured system and clinical reasoning. Conversely, an early symptom code may be unrelated to later heart failure. Definitions, time windows and ascertainment varied, and ejection fraction was often unavailable. Most evidence came from UK health systems. The review was limited to the databases and search terms reported; additional eligible published or unpublished studies may have been missed, potentially affecting the completeness and interpretation of the evidence map.

Causality cannot be established. Hospital-diagnosed patients may have had more severe or rapidly progressive disease, acute precipitating illness or greater comorbidity. Residual confounding remains likely despite adjustment or propensity matching.^4,19,20^ The associations therefore do not prove that earlier investigation would have prevented admission, death or cost. Equally, the absence of validation of a recurrent-presentation trigger should not be taken as evidence that recurrence lacks value; it shows that the proposed pattern has not yet been tested adequately.

### Implications for Practice and Policy

This review does not support a patient-level trigger based solely on recurrence or non-resolution. Its immediate relevance is at pathway level: among patients in whom heart failure was suspected or relevant indicators were recorded, recommended investigation and referral were often not documented. Services can use completion of natriuretic peptide testing, echocardiography and specialist review as auditable measures of pathway performance, while decisions for individual patients remain based on clinical assessment and current guidance.

The findings also point to system constraints. Low recorded use of natriuretic peptide testing and echocardiography across large datasets may reflect access, capacity, coordination and coding as well as clinician decisions.^8-10,14,18^ Associations between hospital-pathway diagnosis and higher mortality or cost support evaluation of services designed to complete diagnostic pathways earlier, but they do not by themselves establish that any particular intervention will improve outcomes.^19^

### Implications for Research

Primary studies should now define repeated presentation explicitly and link patient-level encounters over time. They should distinguish recurrence of the same symptom from evolving symptom clusters, record investigations and referrals, and use blinded clinical adjudication to decide whether an earlier encounter represented no opportunity, a potential opportunity, a missed opportunity or avoidable delay. Analyses should report heart failure phenotype, competing diagnoses, multimorbidity and diagnosis setting, and should validate findings in independent datasets before any electronic trigger is developed.

Only after recurrence shows incremental predictive value beyond age, comorbidity, individual symptoms, prescribing and test results should a candidate indicator be derived and externally validated. Subsequent impact studies would need to test benefits, false-positive investigations, workload, equity and unintended harm. This sequence is more appropriate than moving directly from the present indirect evidence to a clinical alert or prediction model.

## Conclusions

Heart failure is often preceded by symptoms or indicators recorded in primary care, but these observations do not establish that the earliest records represented heart failure or that subsequent intervals were avoidable delays. Investigation and referral were frequently unrecorded or incomplete, and hospital-based diagnosis was common and associated with poorer outcomes. Repeated or unresolved presentation was seldom measured directly; primary studies should now define recurrence, examine clinical responses and adjudicate whether it identifies preventable diagnostic opportunities.

## Supplemental Material

Supplemental Material - Primary Care Signals and Diagnostic Pathways Before Incident Heart Failure: A Systematic Scoping Review Supplemental Material for Primary Care Signals and Diagnostic Pathways Before Incident Heart Failure: A Systematic Scoping Review by Waseem Jerjes, Austen El-Osta, Arnoupe Jhass, Adam Taylor, Gabrielle Oh and Azeem Majeed in Journal of Primary Care & Community Health.

## Data Availability

All data analysed during this study are contained within the published articles included in this review and the supplementary materials accompanying this manuscript. Additional information relating to the study methodology and data extraction is available from the corresponding author upon reasonable request.*

## References

[1] Lawson CA Zaccardi F Squire I , et al. 20-year trends in cause-specific heart failure outcomes by sex, socioeconomic status, and place of diagnosis: a population-based study. Lancet Public Health. 2019;4(8):e406-e420. doi:10.1016/S2468-2667(19)30108-2. 31376859 PMC6686076

[2] Taylor CJ Ordóñez-Mena JM Roalfe AK , et al. Trends in survival after a diagnosis of heart failure in the United Kingdom 2000-2017: population based cohort study. BMJ. 2019;364:l223. doi:10.1136/bmj.l223. Erratum in: BMJ. 2019 Oct 8;367:l5840. doi: 10.1136/bmj.l5840. 30760447 PMC6372921

[3] Koudstaal S Pujades-Rodriguez M Denaxas S , et al. Prognostic burden of heart failure recorded in primary care, acute hospital admissions, or both: a population-based linked electronic health record cohort study in 2.1 million people. Eur J Heart Fail. 2017;19(9):1119-1127. doi:10.1002/ejhf.709. 28008698 PMC5420446

[4] Wang H Gao C Guignard-Duff M , et al. Inpatient versus outpatient diagnosis of heart failure across the spectrum of ejection fraction: a population cohort study. Heart. 2025;111(11):523-531. doi:10.1136/heartjnl-2024-324160. 39880470 PMC12171480

[5] Ali MR Lam CSP Stromberg A , et al. Association of symptoms at heart failure diagnosis with hospitalisation and mortality at 6 and 12 months: a retrospective cohort study using UK primary care health records. BMJ Open. 2026;16(2):e107490. doi:10.1136/bmjopen-2025-107490. PMC12911825 41689226

[6] Hancock HC Close H Fuat A Murphy JJ Hungin AP Mason JM . Barriers to accurate diagnosis and effective management of heart failure have not changed in the past 10 years: a qualitative study and national survey. BMJ Open. 2014;4(3):e003866. doi:10.1136/bmjopen-2013-003866. PMC3975740 24691215

[7] Smeets M Van Roy S Aertgeerts B Vermandere M Vaes B . Improving care for heart failure patients in primary care, GPs' perceptions: a qualitative evidence synthesis. BMJ Open. 2016;6(11):e013459. doi:10.1136/bmjopen-2016-013459. PMC5168518 27903565

[8] Roalfe AK Lay-Flurrie SL Ordóñez-Mena JM , et al. Long term trends in natriuretic peptide testing for heart failure in UK primary care: a cohort study. Eur Heart J. 2021;43(9):881-891. doi:10.1093/eurheartj/ehab781. 34849715 PMC8885323

[9] Taylor CJ Ordóñez-Mena JM Lay-Flurrie SL , et al. Natriuretic peptide testing and heart failure diagnosis in primary care: diagnostic accuracy study. Br J Gen Pract. 2022;73(726):e1-e8. doi:10.3399/BJGP.2022.0278. 36543554 PMC9799346

[10] Zegard A Naneishvili T Viyapurapu R , et al. Diagnostic yield of a heart failure referral pathway using N-terminal pro-brain natriuretic peptide. Open Heart. 2023;10(2):e002469. doi:10.1136/openhrt-2023-002469. 37793674 PMC10551990

[11] Bottle A Newson R Faitna P Hayhoe B Cowie MR . Changes in heart failure management and long-term mortality over 10 years: observational study. Open Heart. 2022;9(1):e001888. doi:10.1136/openhrt-2021-001888. 35354658 PMC8969012

[12] Bottle A Kim D Aylin P Cowie MR Majeed A Hayhoe B . Routes to diagnosis of heart failure: observational study using linked data in England. Heart. 2018;104(7):600-605. doi:10.1136/heartjnl-2017-312183. 28982720

[13] Kim D Hayhoe B Aylin P Majeed A Cowie MR Bottle A . Route to heart failure diagnosis in English primary care: a retrospective cohort study of variation. Br J Gen Pract. 2019;69(687):e697-e705. doi:10.3399/bjgp19X705485. 31455645 PMC6713513

[14] Hayhoe B Kim D Aylin PP Majeed FA Cowie MR Bottle A . Adherence to guidelines in management of symptoms suggestive of heart failure in primary care. Heart. 2019;105(9):678-685. doi:10.1136/heartjnl-2018-313971. 30514731

[15] Conrad N Judge A Canoy D , et al. Diagnostic tests, drug prescriptions, and follow-up patterns after incident heart failure: A cohort study of 93,000 UK patients. PLoS Med. 2019;16(5):e1002805. doi:10.1371/journal.pmed.1002805. 31112552 PMC6528949

[16] Ferreira JP Taveira-Gomes T Canelas-Pais M , et al. Missed opportunities in the diagnosis of heart failure: a real-world assessment. ESC Heart Fail. 2023;10(6):3438-3445. doi:10.1002/ehf2.14531. 37702348 PMC10682848

[17] Migas S Ellis ML Wrona B , et al. Missed opportunities in heart failure diagnosis and management: study of an urban UK population. ESC Heart Fail. 2024;11(4):2200-2213. doi:10.1002/ehf2.14766. 38627992 PMC11287321

[18] Lawson CA Ali MR McCann GP , et al. Health inequalities and trends in heart failure diagnosis in primary care in England, 2000-21: a national retrospective cohort data-linkage study. Lancet Prim Care. 2025;1(6). doi:10.1016/j.lanprc.2025.100060. None. PMC12728148 41451357

[19] Bachtiger P Kelshiker MA Petri CF , et al. Survival and health economic outcomes in heart failure diagnosed at hospital admission versus community settings: a propensity-matched analysis. BMJ Health Care Inform. 2023;30(1):e100718. doi:10.1136/bmjhci-2022-100718. PMC10030479 36921978

[20] Sandhu AT Tisdale RL Rodriguez F , et al. Disparity in the Setting of Incident Heart Failure Diagnosis. Circ Heart Fail. 2021;14(8):e008538. doi:10.1161/CIRCHEARTFAILURE.121.008538. 34311559 PMC9070116

[21] Barber K Bernhardt L McCann GP , et al. Developing core indicators for identifying people at risk of delayed heart failure diagnosis. BMC Prim Care. 2025;26(1):316. doi:10.1186/s12875-025-03024-4. 41102653 PMC12533351

[22] Valk MJ Mosterd A Broekhuizen BD , et al. Overdiagnosis of heart failure in primary care: a cross-sectional study. Br J Gen Pract. 2016;66(649):e587-e592. doi:10.3399/bjgp16X685705. 27266859 PMC4979939

[23] van Riet EE Hoes AW Limburg A Landman MA van der Hoeven H Rutten FH . Prevalence of unrecognized heart failure in older persons with shortness of breath on exertion. Eur J Heart Fail. 2014;16(7):772-777. doi:10.1002/ejhf.110. 24863953

[24] Hawkins NM Scholes S Bajekal M , et al. Community care in England: reducing socioeconomic inequalities in heart failure. Circulation. 2012;126(9):1050-1057. doi:10.1161/CIRCULATIONAHA.111.088047. 22837162

[25] Bottle A Kim D Aylin PP Majeed FA Cowie MR Hayhoe B . Real-world presentation with heart failure in primary care: do patients selected to follow diagnostic and management guidelines have better outcomes? Open Heart. 2018;5(2):e000935. doi:10.1136/openhrt-2018-000935. 30487985 PMC6242017

