# Supplementary material for: Primary Care Signals and Diagnostic Pathways Before Incident Heart Failure: A Systematic Scoping Review
Source: J Prim Care Community Health. 2026 Sep 9;17:21501319261488368. doi: 10.1177/21501319261488368 (PMC13558908; doi:10.1177/21501319261488368)
Supplement: Supplementary file 1 [file sj-pdf-1-jpc-10.1177_21501319261488368.pdf]

Supplementary Appendix 1. Complete electronic search strategies

Primary care signals and diagnostic pathways before incident heart failure: a systematic scoping review

Search scope

Four databases were searched separately on 1 May 2026 for records published from 1 January 2000 to 1 May 2026. The search combined heart failure, primary or ambulatory first-contact care, and prediagnostic pathway concepts. English-language and human-study eligibility criteria were applied, and no study-design filter was used. Counts below are records exported before deduplication.

Database summary

Database	Platform	Coverage and limits	Records
**MEDLINE**	PubMed	1 Jan 2000–1 May 2026; English; humans	88
**Embase**	Ovid	1 Jan 2000–1 May 2026; English; humans	67
**Scopus**	Elsevier Scopus	1 Jan 2000–1 May 2026; English; human eligibility at screening	45
**Web of Science Core Collection**	Clarivate	1 Jan 2000–1 May 2026; English; human eligibility at screening	35
**Total before deduplication**	—	Database exports combined before duplicate removal	235

Search concepts

Concept	Terms represented
**1. Condition**	Heart failure, cardiac failure, congestive heart failure.
**2. Setting**	Primary care, general practice, family practice or family medicine, community care, ambulatory care, outpatient care and first-contact care.
**3. Prediagnostic pathway**	Prediagnostic symptoms or indicators, repeated or unresolved presentation, diagnostic delay, missed opportunity, investigation, natriuretic peptide testing, echocardiography, referral, diagnosis setting and route to diagnosis.
**Symptom expansion**	Breathlessness or dyspnoea, ankle swelling or oedema, fatigue and reduced exercise tolerance were included where supported by the platform syntax.

MEDLINE through PubMed

Line	Search syntax / action
**1**	("Heart Failure"[Mesh] OR "heart failure"[tiab] OR "cardiac failure"[tiab] OR "congestive heart failure"[tiab])

Line	Search syntax / action
**2**	("Primary Health Care"[Mesh] OR "General Practice"[Mesh] OR "Family Practice"[Mesh] OR "Ambulatory Care"[Mesh] OR "Outpatient Clinics, Hospital"[Mesh] OR "primary care"[tiab] OR "general practice"[tiab] OR "general practitioner"[tiab] OR "general practitioners"[tiab] OR "family practice"[tiab] OR "family medicine"[tiab] OR "community care"[tiab] OR "ambulatory care"[tiab] OR "first-contact care"[tiab] OR "first contact care"[tiab] OR outpatient*[tiab])
**3**	("Delayed Diagnosis"[Mesh] OR "Diagnostic Errors"[Mesh] OR "Referral and Consultation"[Mesh] OR "Early Diagnosis"[Mesh] OR "Natriuretic Peptide, Brain"[Mesh] OR "Echocardiography"[Mesh] OR prediagnos*[tiab] OR pre-diagnos*[tiab] OR "diagnostic delay"[tiab] OR "delayed diagnosis"[tiab] OR "delayed diagnoses"[tiab] OR "missed diagnosis"[tiab] OR "missed diagnoses"[tiab] OR "missed opportunity"[tiab] OR "missed opportunities"[tiab] OR "route to diagnosis"[tiab] OR "routes to diagnosis"[tiab] OR "diagnostic pathway"[tiab] OR "diagnostic pathways"[tiab] OR "hospital diagnosis"[tiab] OR "hospital-based diagnosis"[tiab] OR "acute-care diagnosis"[tiab] OR "acute care diagnosis"[tiab] OR "prior symptom"[tiab] OR "prior symptoms"[tiab] OR "symptoms before diagnosis"[tiab] OR "repeated presentation"[tiab] OR "repeated presentations"[tiab] OR "recurrent presentation"[tiab] OR "recurrent presentations"[tiab] OR "unresolved symptom"[tiab] OR "unresolved symptoms"[tiab] OR investigation*[tiab] OR referral*[tiab] OR "natriuretic peptide"[tiab] OR "natriuretic peptides"[tiab] OR echocardiograph*[tiab] OR breathlessness[tiab] OR dyspnea[tiab] OR dyspnoea[tiab] OR "ankle swelling"[tiab] OR edema[tiab] OR oedema[tiab] OR fatigue[tiab] OR "reduced exercise tolerance"[tiab])
**4**	#1 AND #2 AND #3
**5**	#4 AND ("2000/01/01"[Date - Publication] : "2026/05/01"[Date - Publication]) AND English[lang] NOT (animals[mh] NOT humans[mh])
**Result**	88 records exported before deduplication.

Embase through Ovid

Line	Search syntax / action
**1**	exp heart failure/
**2**	(heart failure or cardiac failure or congestive heart failure).ti,ab,kw.
**3**	1 or 2
**4**	exp primary health care/ or exp general practice/ or exp family medicine/ or exp ambulatory care/ or exp outpatient care/
**5**	(primary care or general practice or general practitioner* or family practice or family medicine or community care or ambulatory care or first contact care or first-contact care or outpatient*).ti,ab,kw.
**6**	4 or 5
**7**	exp diagnostic error/ or exp delayed diagnosis/ or exp early diagnosis/ or exp patient referral/ or exp echocardiography/ or exp natriuretic peptide/
**8**	(prediagnos* or pre-diagnos* or diagnostic delay or delayed diagnos* or missed diagnos* or missed opportunit* or route* to diagnosis or diagnostic pathway* or hospital diagnos* or hospital-based diagnos* or acute-care diagnos* or acute care diagnos* or prior symptom* or symptom* before diagnosis or repeated presentation* or recurrent presentation* or unresolved symptom* or investigation* or referral* or natriuretic peptide* or echocardiograph* or breathlessness or dyspnea or dyspnoea or ankle swelling or edema or oedema or fatigue or reduced exercise tolerance).ti,ab,kw.
**9**	7 or 8
**10**	3 and 6 and 9
**11**	limit 10 to human

Line	Search syntax / action
**12**	limit 11 to english language
**13**	limit 12 to yr="2000 - 2026"
**Result**	67 records exported before deduplication on 1 May 2026.

Scopus

Line	Search syntax / action
**1**	TITLE-ABS-KEY(("heart failure" OR "cardiac failure" OR "congestive heart failure") AND ("primary care" OR "general practice" OR "general practitioner" OR "general practitioners" OR "family practice" OR "family medicine" OR "community care" OR "ambulatory care" OR "first-contact care" OR "first contact care" OR outpatient*) AND (prediagnos* OR "pre diagnos*" OR "diagnostic delay" OR "delayed diagnos*" OR "missed diagnos*" OR "missed opportunit*" OR "route to diagnosis" OR "routes to diagnosis" OR "diagnostic pathway*" OR "hospital diagnosis" OR "hospital-based diagnosis" OR "acute-care diagnosis" OR "acute care diagnosis" OR "prior symptom*" OR "symptoms before diagnosis" OR "repeated presentation*" OR "recurrent presentation*" OR "unresolved symptom*" OR investigation* OR referral* OR "natriuretic peptide*" OR echocardiograph* OR breathlessness OR dyspnea OR dyspnoea OR "ankle swelling" OR edema OR oedema OR fatigue OR "reduced exercise tolerance")) AND PUBYEAR > 1999 AND PUBYEAR < 2027 AND (LIMIT-TO(LANGUAGE, "English"))
**Result**	45 records exported before deduplication on 1 May 2026. Human-study eligibility was confirmed during screening.

Web of Science Core Collection

Line	Search syntax / action
**1**	TS=(("heart failure" OR "cardiac failure" OR "congestive heart failure") AND ("primary care" OR "general practice" OR "general practitioner*" OR "family practice" OR "family medicine" OR "community care" OR "ambulatory care" OR "first-contact care" OR "first contact care" OR outpatient*) AND (prediagnos* OR "pre-diagnos*" OR "diagnostic delay" OR "delayed diagnos*" OR "missed diagnos*" OR "missed opportunit*" OR "route* to diagnosis" OR "diagnostic pathway*" OR "hospital diagnos*" OR "hospital-based diagnos*" OR "acute-care diagnos*" OR "acute care diagnos*" OR "prior symptom*" OR "symptom* before diagnosis" OR "repeated presentation*" OR "recurrent presentation*" OR "unresolved symptom*" OR investigation* OR referral* OR "natriuretic peptide*" OR echocardiograph* OR breathlessness OR dyspnea OR dyspnoea OR "ankle swelling" OR edema OR oedema OR fatigue OR "reduced exercise tolerance"))
**2**	Timespan: 1 January 2000 to 1 May 2026; document language: English; Web of Science Core Collection, all subscribed indexes; no document-type or study-design filter.
**Result**	35 records exported before deduplication on 1 May 2026. Human-study eligibility was confirmed during screening.

Supplementary searching and record management

- Reference lists of all included reports and relevant reviews were examined manually for additional eligible records.
- Forward citation searching was undertaken in Scopus and Web of Science Core Collection.
- All exports were combined in the review reference-management workflow before duplicate removal. Database counts therefore represent pre-deduplication exports.
- No study-design filter was applied. Conference abstracts, protocols or registrations without results, records outside the prespecified date range and non-English records were removed before title and abstract screening, as reported in the PRISMA-ScR flow diagram.

Supplementary Appendix 2. Item-level JBI critical appraisal

Primary care signals and diagnostic pathways before incident heart failure: a systematic scoping review

Appraisal approach

Two reviewers independently appraised each included quantitative report using the 11-item JBI cohort checklist used for this review. Items were judged Yes, No, Unclear or Not applicable. Disagreements were resolved through discussion and, where required, a third reviewer. Overall descriptors summarise the pattern and importance of limitations; they were not derived from a numerical cut-off and were not used to exclude reports.

Item key

Item	Appraisal domain (paraphrased)
**Q1**	Comparability of groups and common source population
**Q2**	Consistent exposure classification across groups
**Q3**	Validity and reliability of exposure measurement
**Q4**	Identification of relevant confounding factors
**Q5**	Strategies used to address confounding
**Q6**	Outcome absent at the start of observation or exposure
**Q7**	Validity and reliability of outcome measurement
**Q8**	Follow-up duration reported and sufficient
**Q9**	Follow-up completeness and reasons for loss described
**Q10**	Incomplete follow-up addressed analytically
**Q11**	Statistical analysis appropriate to the design and data

Item-level judgement matrix: Q1-Q6

Report	Q1	Q2	Q3	Q4	Q5	Q6	Overall descriptor
**Bottle et al. 2018 (Heart)**	NA	NA	U	NA	NA	Y	Moderate
**Bottle et al. 2018 (Open Heart)**	N	Y	U	Y	Y	Y	Moderate
**Bottle et al. 2022**	N	Y	U	Y	N	Y	Moderate
**Kim et al. 2019**	N	Y	U	Y	Y	Y	Moderate
**Hayhoe et al. 2019**	NA	Y	U	Y	N	Y	Moderate

Report	Q1	Q2	Q3	Q4	Q5	Q6	Overall descriptor
**Ferreira et al. 2023**	NA	Y	U	Y	U	Y	Moderate
**Migas et al. 2024**	Y	Y	U	Y	Y	Y	Moderate
**Lawson et al. 2025**	N	Y	U	Y	Y	Y	Moderate
**Bachtiger et al. 2023**	Y	Y	U	Y	Y	Y	Moderate
**Wang et al. 2025**	N	N	U	Y	Y	Y	Moderate
**Sandhu et al. 2021**	N	Y	U	Y	Y	Y	Moderate to moderately high

Item-level judgement matrix: Q7-Q11

Report	Q7	Q8	Q9	Q10	Q11	Overall descriptor
**Bottle et al. 2018 (Heart)**	Y	Y	Y	NA	Y	Moderate
**Bottle et al. 2018 (Open Heart)**	Y	Y	Y	Y	Y	Moderate
**Bottle et al. 2022**	Y	Y	Y	Y	Y	Moderate
**Kim et al. 2019**	U	NA	NA	NA	Y	Moderate
**Hayhoe et al. 2019**	U	Y	NA	NA	Y	Moderate
**Ferreira et al. 2023**	U	Y	U	U	Y	Moderate
**Migas et al. 2024**	Y	Y	U	Y	Y	Moderate
**Lawson et al. 2025**	Y	Y	Y	Y	Y	Moderate
**Bachtiger et al. 2023**	Y	Y	Y	Y	Y	Moderate
**Wang et al. 2025**	Y	Y	Y	Y	Y	Moderate
**Sandhu et al. 2021**	U	NA	NA	NA	Y	Moderate to moderately high

Legend: Y, yes; N, no; U, unclear; NA, not applicable. No numerical score or exclusion threshold was used.

Study-specific rationale

Report	Rationale supporting item-level judgements
**Bottle et al. 2018 (Heart)**	Q1, Q2, Q4 and Q5 were not applicable because the principal analysis descriptively reconstructed pathways rather than estimating an exposed-versus-unexposed effect. Q3 was unclear because symptoms, tests and referrals depended on routine coding and could be under-recorded. Incident heart failure was defined after the prediagnostic window (Q6), and linked CPRD, hospital and mortality records provided consistent outcome ascertainment over the reported period (Q7-Q9). Q10 was not applicable because there was no conventional attrition analysis. Descriptive analyses were appropriate to the stated aims (Q11).

Report	Rationale supporting item-level judgements
**Bottle et al. 2018 (Open Heart)**	The management-pathway groups arose from the same data source but were not fully comparable clinically, giving a No judgement for Q1 and a risk of confounding by indication. Pathway classification was applied consistently (Q2), although routine coded management may incompletely represent care (Q3). Important confounders were identified and addressed in adjusted time-to-event analyses (Q4-Q5). Outcomes occurred after symptom presentation and were measured through linked admission and mortality data, with adequate follow-up and censoring methods (Q6-Q10). The statistical approach was appropriate (Q11).
**Bottle et al. 2022**	The two diagnosis eras were not inherently exchangeable because patient mix, coding practice and service organisation changed over time (Q1). The same broad CPRD-based definitions were used across eras (Q2), but exposure measurement remained vulnerable to secular changes and incomplete recording (Q3). Relevant demographic and clinical differences were considered analytically (Q4-Q5). Patients had incident diagnoses and subsequent survival outcomes were obtained from linked records over a sufficient period, with censoring incorporated (Q6-Q10). The comparative and survival analyses were appropriate (Q11).
**Kim et al. 2019**	Patients following and not following the recommended route differed in measured characteristics and practice context (Q1). Route classification was applied consistently (Q2), but depended on routine coding of symptoms, tests and referrals (Q3 and Q7, both Unclear). Patient- and practice-level confounders were identified and addressed using multilevel modelling (Q4-Q5). The route outcome followed the recorded presentation pathway (Q6). Conventional follow-up items were not applicable because the main outcome was route classification at diagnosis (Q8-Q10). The multilevel logistic analysis was appropriate (Q11).
**Hayhoe et al. 2019**	Q1 was not applicable to the principal timing analysis. Symptoms and pathway events were classified using the same code lists across participants (Q2), but first coded symptoms and recorded investigations may not capture all clinical activity (Q3 and Q7, Unclear). Relevant factors affecting investigation, referral and timing were identified and adjusted for (Q4-Q5). The cohort was defined by incident heart failure after the symptom window, and the observation period was sufficient to capture pathway events (Q6-Q8). Linked records and censoring supported follow-up completeness, although unrecorded care remained possible (Q9-Q10). The analyses matched the timing and pathway questions (Q11).
**Ferreira et al. 2023**	The study descriptively assessed patients with a first heart failure hospitalisation, so Q1 was not applicable. Symptom and work-up definitions were applied consistently (Q2), but validity depended on what was recorded within one healthcare organisation and could miss care elsewhere (Q3 and Q7). Potential confounders and clinical characteristics were described (Q4), although the extent of analytic control for confounding was limited (Q5, Unclear). The prehospital window preceded the first admission and was sufficiently long for the stated definition (Q6 and Q8). Completeness of follow-up and handling of care outside the system were not fully established (Q9-Q10). The descriptive and comparative analyses were otherwise appropriate (Q11).
**Migas et al. 2024**	Heart failure phenotype groups were drawn from the same linked regional population using common eligibility criteria (Q1-Q2). Exposure and pathway measurement was partly limited by incomplete natriuretic peptide and ejection-fraction capture (Q3). Relevant comorbidities were identified and incorporated into adjusted survival analyses (Q4-Q5). Incident diagnosis preceded outcomes (Q6), and linked clinical and mortality records supported valid outcome measurement over five years (Q7-Q8). Follow-up completeness was not fully quantified for all data elements (Q9), but censoring and time-to-event methods addressed differing observation time (Q10). Statistical methods were appropriate (Q11).
**Lawson et al. 2025**	Comparison groups defined by period, pathway and patient characteristics were not fully similar at baseline (Q1), although the same national linked-data framework and coding algorithms were applied (Q2). Routine-record indicators and investigations could be under-recorded or misclassified (Q3). A broad set of demographic, clinical and socioeconomic confounders was identified and addressed in adjusted analyses (Q4-Q5). Incident heart failure followed the prediagnostic assessment window, and mortality and diagnosis-setting outcomes were consistently ascertained through linkage with sufficient follow-up (Q6-Q10). The national trend and survival analyses were appropriate (Q11).

Report	Rationale supporting item-level judgements
**Bachtiger et al. 2023**	After propensity-score matching, hospital- and community-pathway groups were balanced on the measured variables used for matching (Q1), and route classification was applied consistently (Q2). The diagnosis-setting exposure was clearly operationalised but could still reflect disease severity or stage, so Q3 was Unclear. Socioeconomic, cardiovascular-risk and prior-resource-use confounders were identified and addressed by matching and adjusted analyses (Q4-Q5). Incident diagnosis preceded mortality and cost outcomes, which were captured through linked records over an adequate period (Q6-Q10). The propensity-matched survival and economic analyses were appropriate (Q11).
**Wang et al. 2025**	Inpatient and outpatient groups differed importantly in baseline severity and clinical context (Q1). In addition, the operational criteria for de novo heart failure differed by diagnosis setting, leading to a No judgement for Q2 and an Unclear judgement for exposure validity (Q3). Relevant confounders were identified and included in adjusted analyses (Q4-Q5). Participants had de novo heart failure and outcomes were measured after diagnosis using linked hospital and mortality records over 365 days (Q6-Q10). The regression and time-to-event analyses were appropriate, while residual severity confounding remained possible (Q11).
**Sandhu et al. 2021**	Acute-care and outpatient diagnosis groups were not clinically comparable at baseline (Q1), although the same claims-based algorithms were applied across groups (Q2). Claims data provide limited symptom and clinical detail, so exposure and diagnosis-setting validity were unclear (Q3 and Q7). Demographic, clinical, socioeconomic and clinician-level factors were identified and addressed in adjusted hierarchical analyses (Q4-Q5). The incident heart failure definition required a prior disease-free period (Q6). Follow-up items were not applicable because the primary outcome was setting of the incident diagnosis rather than a later clinical event (Q8-Q10). Statistical analysis was appropriate, but claims limitations and restricted generalisability supported the higher overall concern (Q11).

Reference for the appraisal instrument

Joanna Briggs Institute. Critical Appraisal Checklist for Cohort Studies. 11-item checklist used in this review; response options: yes, no, unclear and not applicable. The checklist informed appraisal and interpretation but was not converted into a numerical quality score.

-
